# Neuroimaging Markers for Determining Former American Football Players at Risk for Alzheimer's Disease

**DOI:** 10.1089/neur.2022.0020

**Published:** 2022-09-22

**Authors:** Vijaykumar M. Baragi, Ramtilak Gattu, Gabriela Trifan, John L Woodard, Kortney Meyers, Tim S. Halstead, Eric Hipple, Ewart Mark Haacke, Randall R Benson

**Affiliations:** ^1^ Center for Neurological Studies, Dearborn, Michigan, USA.; ^2^ University of Illinois, Chicago, Illinois, USA.; ^3^ Wayne State University, Detroit, Michigan, USA.; ^4^ Subject Recruiter, Fenton, Michigan, USA.; ^5^ HUH-MR Research/Radiology, Wayne State University/Detroit Receiving Hospital, Detroit, Michigan, USA.

**Keywords:** Alzheimer's disease, neuroimaging markers, NFL players

## Abstract

NFL players, by virtue of their exposure to traumatic brain injury (TBI), are at higher risk of developing dementia and Alzheimer's disease (AD) than the general population. Early recognition and intervention before the onset of clinical symptoms could potentially avert/delay the long-term consequences of these diseases. Given that AD is thought to have a long pre-clinical incubation period, the aim of the current research was to determine whether former NFL players show evidence of incipient dementia in their structural imaging before diagnosis of AD. To identify neuroimaging markers of AD, against which former NFL players would be compared, we conducted a whole-brain volumetric analysis using a cohort of AD patients (ADNI clinical database) to produce a set of brain regions demonstrating sensitivity to early AD pathology (i.e., the “AD fingerprint”). A group of 46 former NFL players' brain magnetic resonance images were then interrogated using the AD fingerprint, that is, the former NFL subjects were compared volumetrically to AD patients using a T1-weighted magnetization-prepared rapid gradient echo sequence. The FreeSurfer image analysis suite (version 6.0) was used to obtain volumetric and cortical thickness data. The Automated Neuropsychological Assessment Metric-Version 4 was used to assess current cognitive functioning. A total of 55 brain regions demonstrated significant atrophy or *ex vacuo* dilatation bilaterally in AD patients versus controls. Of the 46 former NFL players, 41% demonstrated a greater than expected number of atrophied/dilated AD regions compared with age-matched controls, presumably reflecting AD pathology.

## Introduction

Alzheimer's disease (AD)—the most common form of dementia—is a neurodegenerative disease characterized by a progressive course of neuronal loss and decline in cognitive, behavioral, and social skills that ultimately result in a person's inability to function independently.^1^ The structural changes in the brain, which begin during the long pre-clinical period (asymptomatic phase), are readily observed with structural neuroimaging.^2–4^ Thus, the use of neuroimaging markers for recognition of these early brain changes, and intervention with disease-modifying drugs before the onset of clinical symptoms, could potentially avert/delay the long-term consequences of this disease. Without a cure or a proven disease-modifying treatment, AD is currently ranked as the sixth-leading cause of death in the United States, with >6 million Americans, most ≥65 years of age, suffering from dementia caused by AD.^5^

Although the etiology of AD is not fully understood, second only to aging, a history of traumatic brain injury (TBI) is the most established environmental risk factor for late-life AD.^6,7^ Repeated mild TBIs (mTBIs), such as those that occur in sports like American football, boxing, hockey, and soccer, are linked to a greater risk of dementia.^8^ A study commissioned by the NFL reported that former players are diagnosed with AD or similar memory-related diseases 19 times the normal rate for men ages 30 through 49.^9^ A brain autopsy study on retired professional football players indicated that a history of repeated TBIs was associated with various neurodegenerative diseases, including AD, chronic traumatic encephalopathy (CTE), Parkinson's disease (PD), and amyotrophic lateral sclerosis (ALS).^10^ Similarly, post-mortem analyses of 35 former professional American football players revealed that 89% of players had CTE, CTE plus AD, or Lewy body disease.^11^ Unfortunately, most of the evidence for AD/dementia in former NFL players is based on post-mortem analysis and represents the late stages of the disease(s).

However, persons with TBI may be in the early, pre-clinical stages of AD without overt symptoms of AD for decades post-injury. There are also data to suggest that TBI may contribute to early-onset AD and may lead to disinhibition, a feature that often results from the frontal effects of TBI.^7^ The long prodrome for AD without overt clinical symptoms post-TBI provides a window of opportunity for detection and intervention early in the disease process. It is in this context that former NFL players, as an “at-risk” population with a history of repetitive TBI and a reported increased incidence of AD, offer a unique opportunity to identify early markers of the disease at a pre-clinical stage that presage a delayed, clinical stage of the disease.

Newer magnetic resonance imaging (MRI) techniques, such as susceptibility weighted imaging, diffusion tensor imaging, magnetic resonance spectroscopy, functional MRI (fMRI), and fluid-attenuated inversion recovery, combined with automated volumetric analysis of three-dimensional (3D) fast low angle shot T1 images, have been shown to be sensitive to changes in the brain induced by typical football collisions.^12^ Moreover, these techniques have proven useful in demonstrating the prognostic value of neuroimaging markers for predicting clinical outcomes such as cognitive deficits in TBI patients.^13,14^ However, currently there are no suitable imaging markers sensitive enough to accurately diagnose the early stages of AD in TBI patients.

Nevertheless, imaging studies in different dementia/AD populations have demonstrated that entorhinal cortex, hippocampus, and amygdala—the limbic regions of brain—are sensitive to early AD pathology.^15–17^ Moreover, the changes in these structures correlate with the overall severity of clinical impairment.^18^ Hippocampus and amygdala subfields have been reported to show differential rates of atrophy early in the disease process (preceding dementia).^19^ Hippocampal subfield segmentation is being used to study early detection of AD, including mild cognitive impairment^20,21^ and AD.^22,23^

Most of the neuroimaging studies conducted so far to diagnose AD have been based on assessment of a limited number of brain structures. However, given the pathological heterogeneity of AD involving multiple brain structures, it is probably impossible to explain AD with a single pathological process.^24,25^ Therefore, the approach in the current study was to perform a comprehensive volumetric analysis on patients diagnosed with AD using all available, atlas-defined brain regions.

This would then allow us to develop an “AD fingerprint,” with the immediate purpose being to identify retired football players who reveal AD-type pathology before diagnosis. Thus, the group of former players in this study, subjected to a comprehensive volumetric neuroimaging analysis and a battery of neuropsychological metrics, was compared with the early-stage AD group using the set of AD-derived predictive brain regions as surrogates of AD pathology. We hypothesized that this approach would increase the specificity of an AD diagnosis and allow for stratification of the former players on their risk for developing AD at some point. The findings from this study could also help with understanding the role of brain trauma as an etiological factor in the pathogenesis of AD. A better understanding of the mechanisms by which trauma increases the likelihood of developing AD could provide important insights regarding strategies for prevention and treatment of the disease.

## Methods

### Subject recruitment and selection

A total of 114 healthy control subjects and 46 former NFL players were included in the study. Former NFL players were recruited from the University of Michigan's Comprehensive Depression Center. A questionnaire was used to obtain information on NFL players regarding the duration of their exposure to tackle football and any concussion symptoms (loss of consciousness, seeing stars, and memory or perceptual impairment) that they recalled along with education and health behaviors, including diet, tobacco use, and alcohol use. The players received a neuropsychological assessment to assess their current level of cognitive functioning. In addition, self-reported post-concussion and/or behavioral/mood symptoms, and responses to an open-text question that was included to capture information on comorbidities and any other diagnosed medical conditions, were recorded.

Healthy control subjects were recruited from Wayne State University and Detroit Medical Center hospital staff, students, and other associated study personnel comprising diverse demographic groups. Flyers posted on the Wayne State campus bulletin board were used to recruit three cohorts of subjects: cohort 1 (*N* = 38), cohort 2 (*N* = 50), and cohort 3 (*N* = 26). A candidate subject with a history of concussion or who played college but not NFL football was excluded. Subjects with cognitive complaints, a diagnosed brain or refractory psychiatric disorder, or illicit drug abuse were also excluded.

The group of former NFL players played in different decades (1960–2010) for varying durations (2–11 years) with a mean of 7 ± 3 (standard deviation; SD) years. Most recalled having concussion symptoms if not a formal diagnosis by a trainer or physician. These players had been referred to the Depression Center for psychiatric evaluation after reporting depression, anxiety, or behavioral issues to the Professional Athletes Foundation and NFL Player Care Foundation. Recruitment and data collection occurred between 2013 and 2016. Players at the time of enrollment were between 29 and 75 years of age with a mean of 48.4 ± 12.2 (SD) years, reflecting a period of 19 ± 11 (mean ± SD) years since last NFL play. Of the 46 players, 80% were Black and 20% White. Their mean education level was 16.1 years (95% confidence interval, 15.7–16.5). None reported using illicit drugs. Most players reported drinking alcohol in moderation.

Control subjects in cohorts 1, 2, and 3 ranged in age from 19 to 57, 19 to 81, and 18 to 60 years, respectively. The pooled group (cohorts 1, 2, and 3 combined) of subjects ranged in age from 18 to 81 years with a mean of 41.4 ± 18.7 (SD) years. They consisted of 55 males and 59 females.

All the participants in the study gave written informed consent in accordance with the Institutional Review Board of Wayne State University (Detroit, MI).

### Magnetic resonance imaging

#### Image acquisition for NFL and control subjects

All study participants were scanned on either 1.5T Sonata or 3T Verio/Trio Siemens systems with a 32-channel head coil (Siemens Medical Solutions USA, Inc., Malvern, PA). T1-weighted 3D magnetization-prepared rapid gradient echo (MPRAGE) scans were acquired for controls and NFL players using the following three image acquisition sequence protocols: Control–cohort 1 (*N* = 38): 3T Verio, repetition time (TR)/echo time (TE) = 1950/2.26 (ms), Sagittal, 0.5 × 0.5 × 1.0 (mm^3^); Control–cohort 2 (*N* = 50); 1.5T Sonata, TR/TE = 800/3.93 (ms), Coronal, 0.75 × 0.75 × 1.50 (mm^3^); and Control–cohort 3 (*N* = 26): 3T Trio, TR/TE = 19/4.92 (ms), Axial, 1 × 1 × 1 (mm^3^); NFL players (*N* = 49): 3T Verio, TR/TE = 2000/3.34 (ms), Axial, 0.67 × 0.67 × 0.70 (mm^3^).

#### Image acquisition for cognitively normal older controls and Alzheimer's disease patients

A group of 59 controls with a mean age of 75.4 ± 6.6 (SD) years and 58 AD patients with a mean age of 75.5 ± 6.6 (SD) years, obtained from the Alzheimer's Disease Neuroimaging Initiative (ADNI) clinical database,^26^ were used for quantitative analysis of brain volumes and cortical thickness. These subjects met the inclusion and exclusion criteria outlined in the ADNI study design protocols.^27^ The subset of control and AD subjects were chosen based on the search criteria matching scanner strength (3T), scanner manufacturer (Siemens), and T1-weighted sequence (MPRAGE), so that the results would reflect differences between groups and not scanner or sequence. A 3D-MPRAGE sequence (T1-MPRAGE) was acquired in the sagittal plane with: flip angle, 9.0 degrees; 1.2-mm isotropic resolution; pulse sequence, gradient recalled/inversion recovery; slice thickness, 1.2 mm; TE, 3.0 ms; inversion time, 900 ms; TR, 2300 ms.

#### Image processing

All T1-MPRAGE scans underwent the same kind of processing for volumetric and cortical thickness analysis using the FreeSurfer (v. 6.0) software package,^28^ as reported previously.^29–31^ All volume and the thickness outputs were produced in text files derived using the “-*recon-all*” flag, which includes motion correction, intensity correction/modulation, intensity normalization, skull stripping, linear registration of images followed by non-linear volumetric registration to Talairach space, smoothing, topology fixation, spherical smoothing, spherical mapping, spherical registration, and cortical parcellation based on the Desikan-Killiany atlas as described elsewhere in numerous publications.^30–33^

A total of 89 regions (34 cortical, 23 subcortical, 22 hippocampal subfields, and 10 amygdalar nuclei) were assessed (see Table 1). For each of the individual regions assessed in the study, volumes of the right (RH) and left hemispheres (LH) were calculated separately. Hippocampal subfield and amygdala subnuclei segmentation was based on a probabilistic brain atlas.^34–37^ All individual volumes and thickness results were age-corrected using the regression slope obtained from the age versus volume plots of the control group.^38^ Once all the individual regional volumes were age-corrected, they were also normalized using total intracranial volume for each subject to remove head-size effects. Amygdala nuclei and hippocampal subfield volumes were normalized to the whole hippocampus and amygdala volumes, respectively, to account for individual differences. Whole hippocampus and amygdala volumes were normalized to intracranial volumes.

**Table 1.** FreeSurfer Parcellation of Brain MR Images of Formal NFL Players, AD Subjects, and Age-Matched Controls

Cortical thickness and volume	Subcortical volume	Hippocampal subfields	Amygdalar nuclei
Bankssts	Lateral-ventricle	Hippocampal tail	Lateral nucleus
Caudal anterior cingulate	Inf-Lat-Vent	Subiculum body	Basal nucleus
Caudal middle frontal	Cerebellum white matter	CA-1 body	Accessory basal nucleus
Cuneus	Cerebellum cortex	Subiculum head	Anterior amygdaloid area
Entorhinal	Caudate	Hippocampal fissure	Central nucleus
Fusiform	Putamen	Presubiculum head	Medial nucleus
Inferior parietal	Pallidum	CA-1 head	Cortical nucleus
Inferior temporal	Hippocampus	Pre-subiculum body	Cortical amygdaloid transition
Isthmus cingulate	Amygdala	Parasubiculum	Paralaminar nucleus
Lateral occipital	Accumbens area	Molecular layer HP head	Whole amygdala
Lateral orbitofrontal	Cortex	Molecular layer HP body	
Lingual	Cerebral white matter	GC-ML-DG-head	
Medial orbitofrontal	Corpus callosum posterior	CA-3 body	
Middle temporal	Corpus callosum midposterior	GC-ML-DG body	
Parahippocampal	Corpus callosum central	CA-4 head	
Paracentral	Corpus callosum midanterior	CA-4 body	
Pars opercularis	Corpus callosum anterior	Fimbria	
Pars orbitalis	Third ventricle	CA-3 head	
Pars triangularis	Fourth ventricle	HATA	
Pericalcarine	CSF	Whole hippocampal body	
Post-central	Brainstem	Whole hippocampal head	
Posterior cingulate	Brain Seg Vol	Whole hippocampus	
Pre-central	Total Gray Vol		
Pre-cuneus			
Rostral anterior cingulate			
Rostral middle frontal			
Superior frontal			
Superior parietal			
Superior temporal			
Supramarginal			
Frontal pole			
Temporal pole			
Transverse temporal			
Insula			

MR, magnetic resonance; AD, Alzheimer's disease; CSF, cerebrospinal fluid; HATA, hippocampal amygdala transition area.

### Assessment of cognitive performance

NFL players were administered the computerized Automated Neuropsychological Assessment Metric Version 4 (ANAM4™) Sports Medicine Battery^39^ and the Wide Range Achievement Test-IV (WRAT-IV) Reading subtest^40^ for assessment of their current cognitive performance and pre-morbid function (pre-morbid IQ estimate), respectively. The ANAM sports medicine battery included the following subtests: simple reaction time; code substitution-learning (CDS); code substitution-delayed (recognition); matching to sample (M2S); mathematical processing; procedural reaction time; spatial processing; Sternberg memory search (ST6); and simple reaction time repeat. Correcting for age and educational level, raw ANAM test scores and WRAT scores were converted to standard scores.

Throughput score, which is the total number of correct responses divided by the time required for those responses, was the primary outcome measure used for each of the ANAM subtests. ANAM throughput Z-scores for individual subtests were used for calculating the correlation (Pearson's coefficient of correlation) between the ANAM subtest throughput scores and the number of atrophied brain regions (>2 SDs from mean of controls). To estimate the change in cognitive ability from pre- to post-injury for a particular player, an average of Z-scores of all the individual ANAM subtests taken by the player was obtained to yield a composite score (average of all ANAM subtest throughput Z-scores) for that player. The composite ANAM Z-score was subtracted from the WRAT Z-score obtained using the raw WRAT score for the player. A negative difference was considered as a decline in the cognitive ability from pre- to post-injury.

### Statistical analysis

Statistical analysis was performed on age-corrected neuroimaging measures of former NFL players and AD subjects. Regression analysis of the corresponding control groups was used to derive age-corrected measures. NFL and AD regional group means were compared to the corresponding control group means to determine percent atrophy or *ex vacuo* dilation. Student's *t*-test was used to assess the significance of the difference between the group means. Values of *p* ≤ 0.05 were considered statistically significant. The data for the right and the left hemispheres were assessed separately.

Only brain regions that demonstrated significant brain atrophy or *ex vacuo* dilatation bilaterally in AD patients were used as imaging markers to probe corresponding brain regions in the former NFL players. The total number of brain regions showing significant atrophy or dilatation (*p* < 0.05) for each former player was compared to the distribution of outliers for controls.

## Results

### Demographics and clinical characteristics of former NFL players

The study cohort of 46 former NFL players was comprised of 80% Black and 20% White athletes, who played in the NFL in different decades (1960–2010) for varying durations (2–11 years) with a mean of 7 ± 3 (SD) years. Players at the time of enrollment were between 29 and 75 years of age with a mean of 48.4 ± 12.2 (SD) years, reflecting a period of 19 ± 11 (mean ± SD) years since last NFL play. Thus, the cohort of NFL players in this study was comprised of subjects who had varying degrees of post-concussion symptoms, including depression, anxiety, irritability, poor memory, difficulty remembering names, and poor concentration. Their neurobehavioral symptom inventory included somatic/sensory (feeling dizzy, loss of balance, and poor coordination), affective (loss of energy, difficulty falling sleep, forgetfulness, and poor concentration), and cognitive symptoms (difficulty making decisions, slowed thinking, and forgetfulness).

In response to an open-text question asking about comorbidities and other medical conditions, whereas most (31%) indicated they had no medical problems, the following comorbidities/other medical conditions were reported by various percentage of players: hypertension (22%); hormone/testosterone deficiency syndrome (6%); adult attention-deficit disorder (ADD; 3%); arthritis (19%); diabetes (3%); attention-deficit hyperactivity disorder (ADHD; 3%); chronic pain/back problem (28%); hyperlipidemia (9%); cancer (3%); and sleep disorder (3%).

### Neuroimaging findings in former NFL players

#### Reduction in cortical thickness and volume

**FIG. 1.**
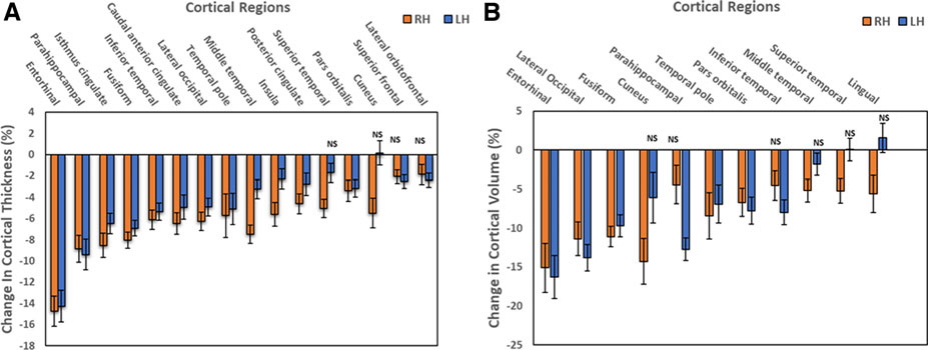
Pattern of regional brain atrophy in NFL players: Cortical regions showing a bilateral reduction in thickness (**A**) and volume loss (**B**) in NFL players compared to controls (*p* < 0.05). Data shown are the mean difference (±SEM) between NFL and control groups expressed as a percentage of controls. Only data that are not statically significant are designated as NS. LH, left hemisphere; RH, right hemisphere; SEM, standard error of the mean.

A total of 114 controls and 46 former NFL players were included in this analysis. Cortical regions demonstrating statistically significant (*p* < 0.05) reduction in thickness or volume in either of the hemispheres of the NFL group compared to controls are shown in Figure 1, panels A and B, respectively. Cortical thinning was most obvious in the medial temporal lobe: entorhinal (RH,14.8%; LH, 14.3%) and parahippocampal (RH, 8.9%; LH, 9.4%); anterolateral temporal lobe: fusiform (RH, 8.1%; LH, 6.9%) and inferior temporal (RH, 6.1%; LH, 5.3%); and in the frontal lobe: isthmus cingulate (RH, 8.5%; LH, 6.5%). The major areas demonstrating loss of cortical volume were in the medial and lateral temporal lobe: entorhinal (RH, 15.1%; LH, 16.3%); parahippocampal (RH, 4.4%; LH, 12.7%); and fusiform (RH, 11.1%; LH, 9.7%); and in the occipital lobe: lateral occipital (RH, 11.4%; LH, 13.8%) and cuneus (RH, 14.3%; LH, 6.1%).

#### Changes in hippocampal subfields and amygdalar nuclei

**FIG. 2.**
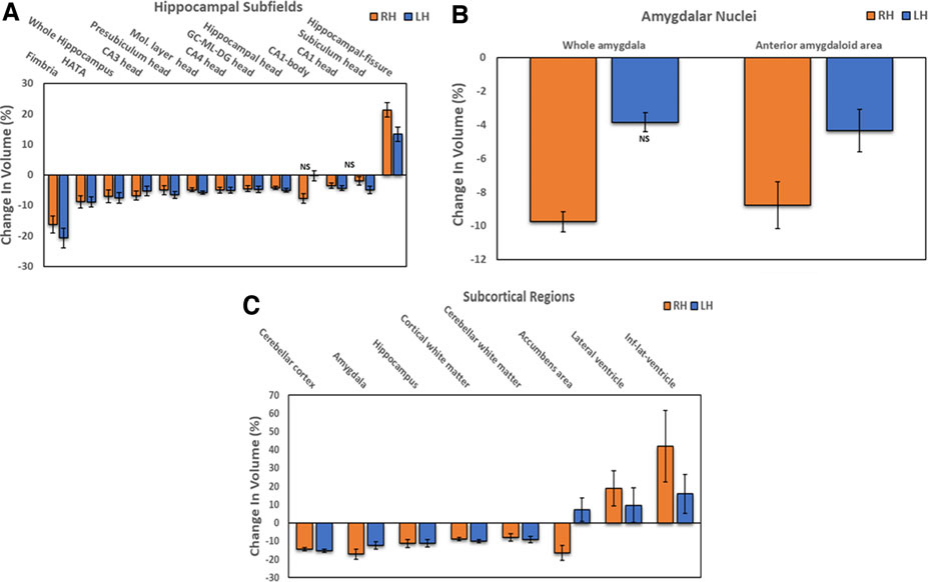
Changes in limbic structures and subcortical regions in NFL players: bilateral loss in volume of hippocampal subfields (**A**), amygdalar nuclei (**B**), and subcortical regions (**C**) in NFL players compared to controls (*p* < 0.05). Data shown are the mean difference (±SEM) between NFL and control groups expressed as a percentage of controls. Only data that are not statically significant are designated as NS. LH, left hemisphere; RH, right hemisphere; SEM, standard error of the mean.

Hippocampal subfields and amygdalar nuclei have been reported to show different rates of atrophy early in the neurodegenerative disease process (preceding dementia). Therefore, to examine potential changes in the hippocampus and amygdala, subfield segmentation analysis was performed on brain images of former NFL players in comparison with images from 88 controls. Hippocampal subfields and amygdalar nuclei demonstrating statistically significant (*p* < 0.05) change in either of the hemispheres of the NFL group are shown in Figure 2, panels A and B, respectively. Fimbria (RH, 16.3%; LH, 20.7%) followed by the hippocampal amygdala transition area (HATA; RH, 8.9%; LH, 8.9%) were the most affected of the hippocampal subfields examined. Whole hippocampus volume, representing the sum of all the subfields, was also significantly reduced compared to the control group (RH, 7.0%; LH, 7.5%). In contrast, there was a significant compensatory enlargement of hippocampal fissure, the laterally localized hippocampal cavity (RH, 21.3%; LH, 13.3%). The anterior amygdaloid area (RH, 8.8%; LH, 4.3%) and whole amygdala volume (RH, 9.8%; LH, 3.9%) were the only amygdalar regions demonstrating significant atrophy in the NFL group.

#### Changes in subcortical regions

Figure 2C depicts the subcortical regions with statistically significant (*p* < 0.05) changes in volume in hemispheres of the NFL group compared to the control group. A total of 114 controls and 46 former NFL players were included in this analysis. Cerebellar cortex (RH, 14.3%; LH, 15.4%), amygdala (RH, 17.1%; LH, 12.4%), and hippocampus (RH, 11.4%; LH, 11.2%) showed the most pronounced difference in volumes between the NFL and control groups, whereas the inferior-lateral ventricle (RH, 42.0%; LH, 15.9%) and lateral ventricle (RH, 18.8%; LH, 9.6%) demonstrated significant increases in their volume.

### Neuropsychological performance of former NFL players

There was a high degree of intrasubject intertask correlation across the ANAM throughput measures (data not shown). Most of the NFL players subjected to neuropsychological assessment demonstrated poor performance on most of the ANAM subtest scores, as indicated by their negative Z-throughput scores. This decline in neuropsychological performance was also evident when the composite ANAM Z-scores (average of all the ANAM subtest throughput Z-scores) were compared to the respective WRAT Z-scores (pre-morbid estimate). Of the 42 NFL players subjected to neuropsychological assessment, 23 (55%) demonstrated a decline in cognitive ability from pre- to post-injury. Decline in neuropsychological parameters also correlated with neuroimaging changes, as indicated by the number of atrophied brain regions in NFL players (Table 2). Players with a higher number of atrophied brain regions showed poorer performance on ANAM subtests. ANAM subtests that showed significant correlation (*p* < 0.05) with the number of atrophied brain regions were M2S and CDS, with Pearson's correlation coefficient (*r*) of −0.35 and −0.31, respectively (Table 2).

**Table 2.** Correlation between ANAM Subtest Scores and the Number of Atrophied Brain Regions of Former NFL Players (*N* = 41)

ANAM subtests	Correlation (*r*)	*p* value
CDD: code substitution-delayed (recognition)	**–0.26**	**<0.10**
CDS: code substitution-learning	**–0.31**	<0.04^**^
M2S: matching to sample	**–0.35**	<0.02^**^
MTH: mathematical processing	**–0.11**	**<0.50**
PRO: procedural reaction time	**–0.11**	**<0.49**
SPD: spatial processing	**–0.13**	**<0.41**
SR2: simple reaction time repeat	**–0.23**	**<0.15**
SRT: simple reaction time	**–0.12**	**<0.43**
ST6: Sternberg memory search	**–0.18**	**<0.25**
Composite: average of ANAM subtests	**–0.25**	**<0.11**

Data shown are Pearson's correlation coefficients between ANAM subtest Z-throughput scores and the number of atrophied brain regions (>2 SDs from mean of controls) in the former NFL players.

^**^ Statistically significant *p* values.

ANAM, Automated Neuropsychological Assessment Metric; SD, standard deviation.

### Neuroimaging findings in Alzheimer's disease subjects

#### Alzheimer's disease fingerprint

**FIG. 3.**
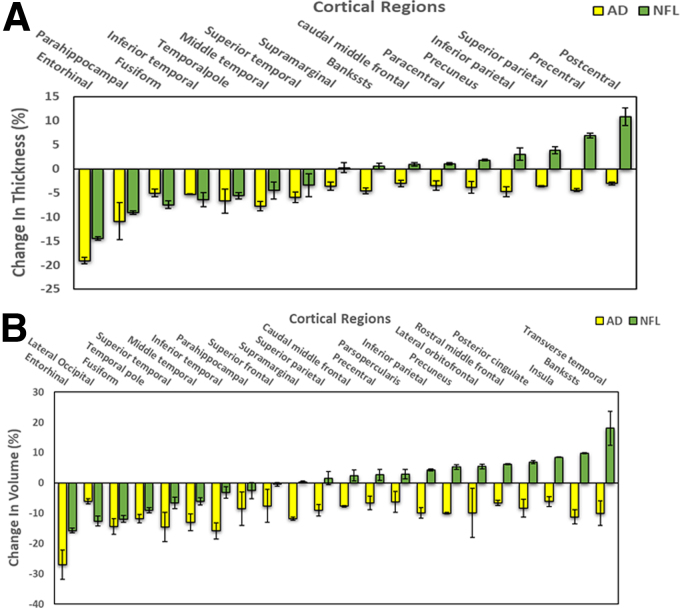
“AD fingerprint” of regions prone to atrophy or *ex vacuo* dilatation in AD subjects and corresponding atrophied/dilated regions in NFL players. Data shown are mean (±SEM) of right and left hemispheric reduction in cortical thickness (**A**) and volume loss (**B**). All the mean differences between AD and their age- and sex-matched control group (yellow bars) and NFL players and their control group (green bars), expressed as a percentage of controls, are statistically significant (*p* < 0.05). AD, Alzheimer's disease; SEM, standard error of the mean.

**FIG. 4.**
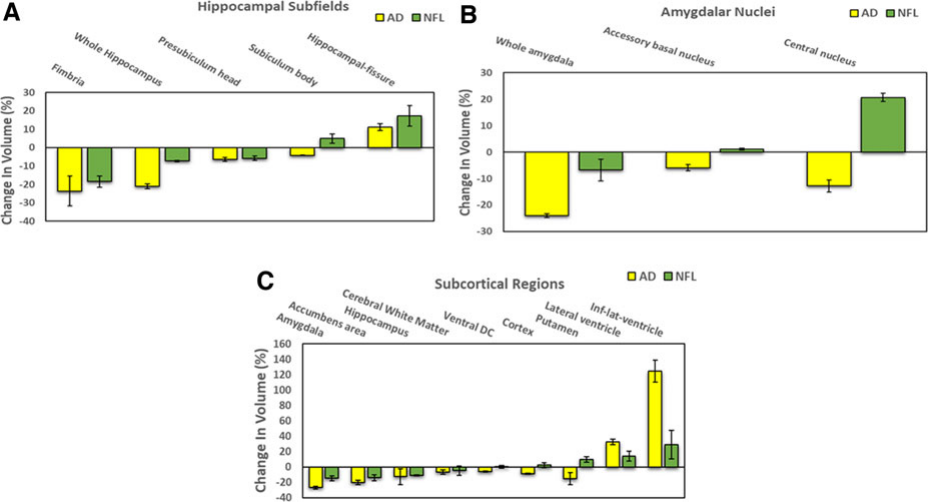
Changes in limbic structures and subcortical regions of AD subjects compared with NFL players. Data shown are mean (±SEM) of right and left hemispheric reduction in volume of hippocampal subfields (**A**), amygdalar nuclei (**B**), and subcortical regions (**C**) in AD subjects and NFL players compared to their respective controls (*p* < 0.05). All the mean differences between AD and their age- and sex-matched control group (yellow bars) and NFL players and their control group (green bars), expressed as a percentage of controls, are statistically significant (*p* < 0.05). AD, Alzheimer's disease; SEM, standard error of the mean.

To develop a “fingerprint” of regions prone to atrophy or *ex vacuo* dilatation in AD, a volumetric analysis of all brain regions of patients diagnosed with early AD was performed against age-matched controls. A total of 59 controls and 58 AD subjects diagnosed with early-stage AD were included in this analysis. Means of RH and LH brain regions that demonstrated bilaterally significant differences (*p* < 0.05) in thickness or volume in AD subjects compared to their age- and sex-matched controls (24 cortical, 9 subcortical, 5 hippocampal subfields, and 3 amygdalar nuclei) are shown in Figures 3 and 4.

#### Cortical regions

Cortical regions demonstrating statistically significant (*p* < 0.05) reduction in thickness or volume bilaterally in the AD group compared to controls are shown in Figure 3, panels A and B, respectively. The temporal lobe was the most affected area of the brain of AD subjects with the greatest number (10 regions) and extent of atrophied regions, followed by parietal (seven regions), frontal (six regions), and occipital lobes (one region). Severity of injury, in terms of the degree or extent of brain tissue damage within the temporal lobe, was in the medial temporal lobe: entorhinal (thickness, 19.1%; volume, 27%), parahippocampal (thickness, 10.9%; volume, 8.5%), and in the anterolateral temporal lobe: fusiform (thickness, 5.1%; volume, 14.5%), middle temporal (thickness, 7.8%; volume, 13.0%), inferior temporal (thickness, 5.3%; volume, 15.8%), superior temporal (thickness, 6.0%; volume, 14.5%), and temporal pole (thickness, 6.7%; volume, 11.9%).

The areas most affected in the parietal lobe were: supramarginal (thickness, 3.6%; volume, 11.8%), superior parietal (thickness, 3.6%; volume, 9.0%), pre-cuneus (thickness, 3.9%; volume, 10%), and inferior parietal (thickness, 4.8%; volume, 9.9%); frontal lobe: lateral orbitofrontal (volume, 10%), caudal middle frontal (thickness, 3.0%; volume, 7.7%), and superior frontal (volume, 7.6%).

#### Hippocampal subfields and amygdalar nuclei

Hippocampal subfields and amygdalar nuclei demonstrating statistically significant (*p* < 0.05) differences with controls bilaterally are shown in Figure 4, panels A and B, respectively. Whereas fimbria (23.7%), whole hippocampus (21.1%), and whole amygdala (24.0%) were the most atrophied regions, hippocampal fissure (11.2%) alone demonstrated *ex vacuo* dilatation. However, pre-subiculum head, subiculum body in the hippocampus, and accessory basal nucleus and central nucleus in the amygdala were also significantly reduced in volume for AD compared to the controls.

#### Subcortical regions

Figure 4C depicts the subcortical regions with statistically significant (*p* < 0.05) changes in volume bilaterally in the AD group compared to the control group. Amygdala (26.8%), nucleus accumbens (20.0%), and hippocampus (12.5%) were the most atrophied subcortical regions. On the other hand, volumes of inferior-lateral ventricle (124.7%), lateral ventricle (32.5%), and putamen (15.3%) were significantly enlarged in AD.

### Relevance of the neuroimaging findings in former NFL players to AD brain changes

The set of regions demonstrating bilaterally significant brain atrophy or *ex vacuo* dilatation in AD patients was used to interrogate the structural brain images of former NFL players for the purpose of identifying changes in their brains indicating likely AD pathology.

#### Cortical regions

The greatest number and magnitude of atrophied cortical regions in former NFL players corresponding to affected areas in AD subjects were in the anterolateral and mesial areas of the temporal lobe (Fig. 3A,B): entorhinal, parahippocampal, middle temporal, inferior temporal, superior temporal, and temporal pole. Lateral occipital volume was also significantly reduced in both NFL and AD subjects.

#### Hippocampal subfields and amygdalar nuclei

In both NFL and AD subjects, volumes of fimbria, pre-subiculum head, whole hippocampus, and whole amygdala were greatly reduced, whereas hippocampal fissure volume was dilatated in both (Fig. 4A,B).

#### Subcortical regions

Whereas whole amygdala, nucleus accumbens, and whole hippocampus were the most atrophied subcortical regions for both NFL and AD, volumes of inferior-lateral ventricle and lateral ventricle were significantly enlarged in both AD and NFL subjects (Fig. 4C).

### Rate of Alzheimer's disease pathology in former NFL players

**FIG. 5.**
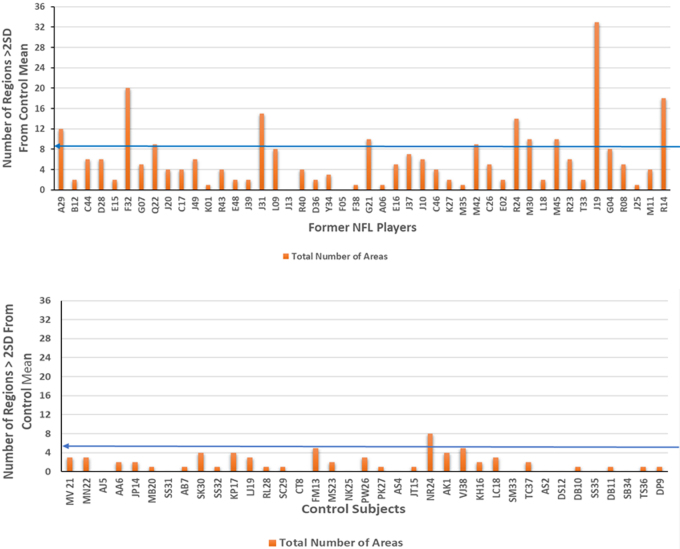
Subjects with presumed AD pathology in the NFL cohort. Shown are subjects in the NFL (A) and control groups (B) with a greater than expected number of atrophied/dilated AD regions. The blue line with the arrowhead demarcates the number of subjects with a greater than expected number of atrophied/dilated regions based on the cutoff value of 5.44 (control mean ± 2 SDs). AD, Alzheimer's disease; SDs, standard deviations.

For each former NFL player, the number of regions showing significant change (compared with corresponding controls) in either hemisphere (*p* < 0.05) was used as a potential marker of AD pathology. Shown in Figure 5A,B are the number of regions with presumptive AD pathology (i.e., >2 SDs from the mean number for the controls = 1.78) for each of the NFL players and control subjects, respectively. These regions are used as a fingerprint of early-stage AD, demonstrating bilaterally significant brain atrophy or *ex vacuo* dilatation in our 58 AD subjects. Using a threshold of 5.44 regions (mean number for the controls +2 SDs), 19 of 46 (41%) former NFL players were identified as having significantly more AD-type atrophy than controls.

### Effect of age and disease on neuroimaging changes in former NFL players

**FIG. 6.**
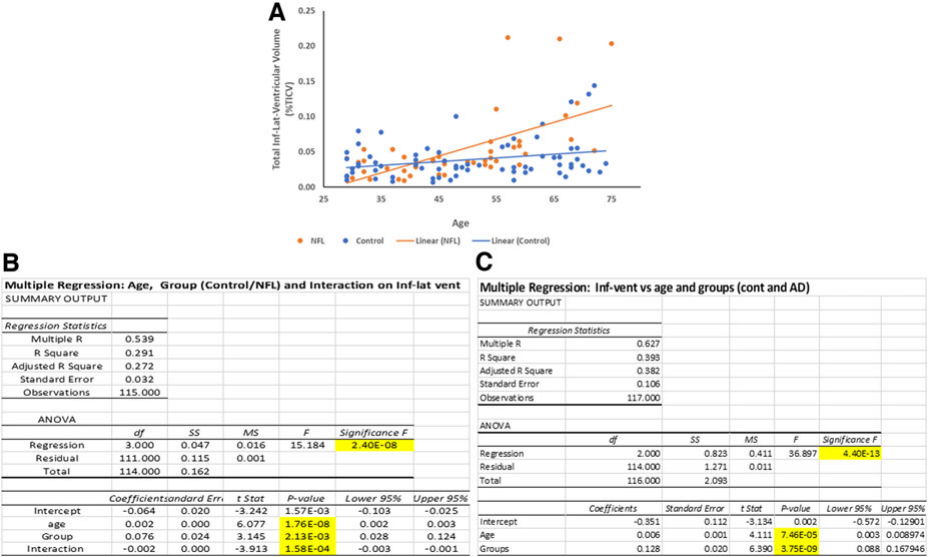
Effect of age, disease, and their interactions on inferior-lateral ventricular volume in NFL, AD, and control subjects. Age has an accelerated effect on inferior lateral-ventricular volume among NFL players compared to control subjects (**A**). Multiple regression analysis demonstrated that accelerated increase in the volume of inferior lateral-ventricular among NFL players was a result of combined effect of age, group (control vs. NFL), and their interactions, with each predictor (age, group, and age-group interaction) having a significant effect (*p* < 0.05) (**B**). Multiple regression analysis of factors contributing to increased volume among AD subjects compared to their controls also indicated that both age and group/disease (control vs. AD) have a significant effect on inferior lateral-ventricular volume (*p* < 0.05) (**C**). AD, Alzheimer's disease; ANOVA, analysis of variance.

Multiple regression analysis of age, group/disease (i.e., control and NFL group), and their interaction on various neuroimaging metrics, including hippocampus, amygdala, entorhinal, fusiform, inferior-lateral ventricle, and lateral ventricular volume, was assessed in both AD and NFL groups in comparison with their respective controls. There were significant (*p* < 0.05) changes in the hippocampal, amygdala, fusiform, inferior lateral-ventricle, and lateral ventricular volume with age in the NFL group; however, only the inferior-lateral ventricle and lateral ventricular volumes showed a significant increase in volume with age in the control group (data not shown). Inferior-lateral ventricular volume increased with age much more rapidly in the NFL group than in the control group (Fig. 6A). The inferior-lateral ventricular volume increase in the NFL group was significantly (*p* < 0.05) affected by age, group/disease, as well as interaction between age and group/disease (Fig. 6B). Lateral ventricular volume was also found to increase significantly (*p* < 0.05) with age and disease in the AD group compared to its control group (Fig. 6C).

## Discussion

NFL players, presumably by virtue of their exposure to repetitive TBI, are at a higher risk of developing AD and other neurodegenerative diseases later in life than the general population.^9^ However, neuroimaging may provide biomarkers in neurological conditions, such as AD, well before clinical symptoms are fully apparent or before irreversible neuronal damage has occurred.^2–4^ The aim of the current research was to identify neuroimaging biomarkers of AD, against which retired American football players' MRIs would be compared, to identify persons at increased risk of developing AD.

Our approach was, first, to develop an AD fingerprint by comparing a group of early-stage AD patients to age- and sex-matched healthy controls using volumetric analysis on T1-weighted MRIs. The second step was to characterize the volumetric differences between a cohort of former NFL players and a group of age- and sex-matched controls. The third step was to apply the AD fingerprint to results for former NFL subjects obtained in the second step to identify atrophied/dilated regions in former NFL subjects corresponding to the AD fingerprint.

### Brain structural changes in early-stage Alzheimer's disease

The temporal lobe was the most affected portion of the cerebrum in AD subjects, followed by parietal, frontal, and occipital lobes, compared to age-matched controls (Fig. 3A,B). Severity of atrophy within the temporal lobe was greatest in the medial temporal lobe: entorhinal (thickness, 19.1%; volume, 27%), and parahippocampal (thickness, 10.9%; volume, 8.5%). These findings are consistent with several previous MRI studies in AD that have demonstrated that the medial temporal lobe, particularly the entorhinal cortex and hippocampus, are severely reduced in size even before the time patients are mildly affected clinically.^41–46^ Neuropathological changes in the medial temporal lobe, including reduction in volumes of the amygdaloid complex, hippocampal formation, subiculum, and parahippocampal cortex, are believed to be the earliest and most prominent features of disease contributing to memory impairment in AD patients.^47^ It is reported that medial temporal lobe atrophy is a reliable marker for identifying patients with AD.^48–51^

AD subjects in the present study also demonstrated significant atrophy in several regions of the anterolateral temporal lobe, including fusiform (thickness, 5.1%; volume, 14.5%), middle temporal (thickness, 7.8%; volume, 13.0%), inferior temporal (thickness, 5.3%; volume, 15.8%), superior temporal (thickness, 6.0%; volume, 14.5%), and temporal pole (thickness, 6.7%; volume, 11.9%). Whereas medial temporal lobe atrophy is well described in AD, atrophy of the anterior temporal lobe is not specific to AD; it is also observed in semantic dementia.^52,53^ However, serial brain MRI measurements of AD subjects indicate that rates of atrophy vary with the stage of the disease, ^45^ and that the greatest rates of atrophy are observed in the inferior and lateral temporal lobe cortices of subjects with moderately severe AD.^45^

The fimbria (23.7%), whole hippocampus, (21.1%), and whole amygdala (24.0%) were the most atrophied limbic regions in AD in the present study (Fig. 4A,B). However, only the hippocampal fissure (11.2%), which separates the dentate from the subiculum, demonstrated *ex vacuo* dilatation (Fig. 4A). The amygdala, which, along with the hippocampus, has a major role in the consolidation of emotional memory,^54^ has been reported to show prominent atrophy early in the disease process and has been found to correlate with symptom severity.^18^ Similar to our findings, it has been reported that hippocampal volume was significantly lower, and inferior lateral ventricular volume significantly higher, in patients with AD than in healthy controls, suggesting that hippocampal and inferior lateral ventricular volumes in combination can be used in the differential diagnosis of AD.^55^

Although it has been reported that subfield analysis of the medial temporal lobe may enhance the predictive value for the diagnosis of AD,^56^ there has been no consensus as to which subfield is a reliable, early-stage disease biomarker. The CA1, entorhinal cortex, subiculum, and CA1-2 transition zones have been reported to be reduced in size in AD patients.^56^ In the current study, the fimbria, pre-subiculum head, and subiculum body in the hippocampus, and accessory basal and central nuclei in the amygdala, were the only subfields that were significantly reduced in volume in AD subjects.

Historically, AD has been characterized mainly by atrophy of the cortex. However, recent imaging studies indicate that alterations in subcortical structures may be of value for early detection of AD.^21^ Various subcortical regions, including nucleus accumbens, amygdala, caudate nucleus, hippocampus, pallidum, putamen, and thalamus—associated with memory, emotional learning, shifting attention, and spatial working memory—have been reported to be typically impaired in AD.^57,58^ More recently, it has been reported that the atrophy in the hippocampus and nucleus accumbens was associated with cognitive impairment in AD patients,^59^ and that these regions could potentially be targeted for treatment of AD.^59^ Consistent with these reports, amygdala, nucleus accumbens, cerebral white matter, putamen, and hippocampus were found to be atrophied in AD patients in the current study. Volumes of the inferior lateral ventricle and lateral ventricle, on the other hand, were significantly enlarged in AD (Fig. 4C).

### Brain structural changes in former NFL players

To characterize the brain structural differences in former NFL players, cortical and subcortical regions, including limbic (hippocampal and amygdala subfields) regions, of former NFL players were compared with those of matched healthy control subjects. Cortical changes of significance in NFL players were cortical thinning and volume loss. The most affected regions of the cortex were medial and anterolateral temporal lobe, followed by frontal and occipital lobes (Fig. 1A,B).

Although the volumes of both the hippocampus and amygdala were reduced, the subfields that demonstrated maximum volume loss were fimbria and HATA in the hippocampus and the anterior amygdaloid area in the amygdala (Fig. 2A,B). In contrast, the hippocampal fissure, the laterally located hippocampal cavity, was significantly enlarged (Fig. 2A), attributable to hippocampal atrophy. Increased hippocampal fissure width has been reported to be a sensitive indicator of hippocampal atrophy in rats.^60^ Subcortical structures that showed pronounced differences in atrophy between the NFL and control groups were amygdala, hippocampus, and cerebellar cortex, whereas inferior lateral ventricle and lateral ventricle demonstrated substantially increased volume in NFL players compared to controls (Fig. 2C).

To confirm that the regional differences obtained for the former NFL players were not simply an effect of aging, multiple regression, with age and group as predictors, was performed. The results, as shown for inferior lateral ventricular volume, indicate that whereas age was a main effect, there was also a significant effect of group (former NFL vs. controls) and an even stronger effect of interaction between age and group on inferior lateral ventricular volume. This indicated a differential (i.e., a greater) effect of age on the former NFL group compared with controls. In other words, there appeared to be a greater effect of aging on brains of former NFL subjects compared with controls, leading to regionalized atrophy (Fig. 6A,B).

There are limited neuroimaging data on sports-related TBIs. Most studies, especially those involving former NFL players, have focused on the assessment of TBI-related neuropsychiatric symptoms^9,61–63^ and diagnosis of putative CTE.^9,11,61–64^ Available neuroimaging data are limited to autopsy studies on brains of former football players,^11,64,65^ and a pilot imaging study on 9 former NFL players and their 9 age-matched controls.^66^ These studies were primarily focused on the diagnosis of putative CTE—characterized by global brain atrophy, including the cerebral hemispheres, medial temporal lobe with ventricular dilatation, and a fenestrated cavum septum pellucidum—and involved only a limited number of subjects. The current study, on the other hand, studied a larger cohort of former NFL players without a diagnosis of dementia, comparing regional brain structure to that of healthy controls, which differed by exposure to brain trauma.

### Comparison of neuropsychological and neuroimaging changes in former NFL players

Neuropsychological decline resulting from TBI in tackle football can herald the onset of AD/dementia after TBI. Assessing neuropsychological findings in relation to neuroimaging biomarkers in persons who show decline can be potentially useful in assessing the risk for neurodegenerative diseases such as AD.^67,68^ Thus, we assessed the relationship between ANAM subtest scores and the number of atrophied brain regions in NFL players. Whereas all ANAM subtest scores correlated negatively with the number of atrophied brain regions, the ANAM subtests that showed a significant correlation (*p* < 0.05) with the number of atrophied brain regions were M2S and CDS, with a Pearson's correlation coefficient (*r*) of −0.35 and −0.31, respectively (Table 2). Interestingly, AD patients were reported to perform worse than controls on matching to sample (M2S), running memory continuous performance test, and Sternberg six-letter memory (ST6), indicating working memory impairments in AD subjects.^69^

An fMRI resting-state functional connectivity study of mTBI subjects reported that reduced resting-state functional connectivity may contribute to cognitive deficits in high-symptom mTBI participants. The study reported that compared to control subjects and low-symptom mTBI participants, high-symptom mTBI participants performed poorly on code substitution–delayed, code substitution, and repeated simple reaction time ANAM subtests and demonstrated reduced interhemispheric functional connectivity and cognitive performance.^70^ Similar to these findings, code substitution (*r* = −0.31, *p* < 0.05), code substitution–delayed (*r* = −0.26, *p* < 0.1) and repeated simple reaction time (*r* = −0.23, *p* < 0.15) showed a higher correlation with brain atrophy than other ANAM subtests evaluated in the current study.

### Comparison of structural changes in former NFL players to Alzheimer's disease

Given the reported increased risk of AD/dementia after TBI, especially among former NFL players who were exposed to repetitive TBI,^9^ and the lack of reported neuroimaging data in former NFL players as it relates to AD, we interrogated the brain images of former NFL players utilizing an AD fingerprint derived from the first step, that is, structural differences between early-stage AD and healthy controls.

The greatest number and the extent of atrophied cortical regions in the former NFL players corresponding to the affected areas in AD subjects were in the anterolateral and medial areas of the temporal lobe (Fig. 3A, B): entorhinal, parahippocampal, middle temporal, inferior temporal, superior temporal, and temporal pole demonstrated severe cortical thinning and volume loss in both former NFL players and AD patients. Entorhinal was the most severely affected area with volume loss of 27% and 16%, and cortical thinning of 19% and 14.5% in AD patients and former NFL players, respectively. Next to entorhinal, parahippocampal was the area that demonstrated maximum cortical thinning of ∼10% in both former NFL players and AD patients, respectively. The middle temporal, inferior temporal, superior temporal, and temporal pole exhibited significant reductions in volume ranging from 6% to 12% and 12% to 16% in former NFL players and AD patients, respectively. Amygdala, nucleus accumbens, cortical white matter, and hippocampus were also found to be atrophied in both NFL players and AD patients in the current study. The volume of lateral occipital cortex—an area consistently atrophied in AD patients,^71^ was also markedly reduced in both NFL and AD subjects.

In the current study, the fimbria and pre-subiculum head were the only hippocampal subfields that were significantly reduced in volume for both NFL and AD subjects, whereas hippocampal fissure volume was demonstrably dilated (Fig. 4A,B).

Volumes of lateral and inferior-lateral ventricle were significantly increased for both AD and NFL subjects (Fig. 4C). As discussed earlier, both age and disease correlated with ventricular volume, especially inferior-lateral ventricular volume (Fig. 6A–C).

Cortical changes, along with the marked reduction in the volumes of hippocampus and amygdala (Fig. 4A–C), suggest that changes in the temporal lobe, particularly those in the medial temporal lobe of former NFL players, reflect AD pathology and have potential utility in identifying former NFL players at risk for developing serious structural and functional changes associated with AD.

### Prevalence and potential risk factors of AD in former NFL players

As discussed earlier, NFL players, by virtue of their exposure to repetitive TBI, are at higher risk of developing AD/dementia than the general population.^9^ Therefore, the players in this study, who were recruited from the Professional Athletes Foundation and NFL Player Care Foundation, were assessed for AD prevalence and risk. Although these players had been referred to the Depression Center for psychiatric evaluation after reporting depression, anxiety, or behavioral issues, they were not specifically evaluated by the Depression Center for AD. Therefore, at the time of enrollment in our study, none of the players were diagnosed or treated for AD. However, given that the risk of developing neurodegenerative disease later in life is reported to increase with severity and frequency of trauma,^72^ we assessed the percentage of former NFL players with a greater than expected number of atrophied/dilated AD regions compared with age-matched controls to estimate the prevalence of presumed AD pathology in the NFL cohort. Of the 46 former NFL players, 19 (41%) demonstrated a greater than expected (>2 SDs from the mean of controls) number of atrophied/dilated AD regions (Fig. 5A,B).

The remarkably high proportion of former NFL players with AD-like pathology may reflect the demographics and clinical characteristics of the patient population and may underlie the neuropsychiatric symptoms reported by these subjects at the time of enrollment in the study. Study participants were highly heterogeneous in terms of age, medical history, number of comorbidities, length of NFL career, concussion history, and neuropsychiatric symptoms.

Players at the time of enrollment were between 29 and 75 years of age, reflecting a period of 19 ± 11 (mean ± SD) years since last NFL play. They had played in the NFL in different decades (1960–2010) and for varying durations of time (2–11 years). Thus, the cohort of NFL players in this study was comprised of subjects who had varying degrees of post-concussions symptoms, including depression, anxiety, irritability, poor memory, difficulty remembering names, and poor concentration. Their neurobehavioral symptom inventory included somatic/sensory (feeling dizzy, loss of balance, and poor coordination), affective (loss of energy, difficulty falling sleep, forgetfulness, and poor concentration), and cognitive symptoms (difficulty making decisions, slowed thinking, and forgetfulness).

Players had medically or family-member–documented histories of treatment for various comorbidities, including progressive cognitive, psychiatric, and/or motor symptoms. They were, however, not diagnosed or treated for AD. Although most players (31%) indicated that they had no medical problems, varying comorbidities/other medical conditions were reported by various players: hypertension (22%); hormone/testosterone deficiency syndrome (6%); adult ADD (3%); arthritis (19%); diabetes (3%); ADHD (3%); chronic pain/back problems (28%); hyperlipidemia (9%); cancer (3%); and sleep disorders (3%). However, given that the subjects were not evaluated for AD when referred to the Depression Center for psychiatric evaluation, none of the players in the study were diagnosed or treated for AD at the time of enrollment.

Given that these players had played for an average of 7 ± 3 (SD) years, it is possible that the long duration of play and the potential repeated TBIs suffered during these years could also have contributed to the higher percentage of players with AD-like pathology. Though there are no data correlating the duration of play and subsequent development of AD, it has been well documented that the odds of developing CTE, a neurodegenerative disease, doubles every 2.6 years of play.^73^ In addition to the long duration of play, there was a gap of 19 ± 12 (mean ± SD) years between time of last NFL play and the neuroimaging and neuropsychological assessment. Given that a remote history of TBI is believed to elevate the risk of developing AD and lower the age of onset of AD,^74^ the post-concussion and neurobehavioral symptoms and the higher rates of AD-like pathology observed are possibly attributable to the long-term consequences of TBI experienced by the former NFL players in the current study.

Consistent with our study, recent NFL concussion data suggested that nearly 30% of former NFL players will develop AD or dementia.^75^ A brain autopsy study on retired professional football players indicated that a history of repeated TBIs was associated with various neurodegenerative diseases, including AD, CTE, PD, and ALS.^10^ A study commissioned by the NFL reported that former players appear to be diagnosed with AD or similar memory-related diseases 19 times the normal rate for men ages 30 through 49.^9^ Taken together, these findings attest to the high risk of developing AD/dementia associated with high-impact collisions experienced by former NFL players. However, as is the case with many cross-sectional studies, the neuroimaging and neuropsychological data in this study were collected at a single time point and therefore represent only a snapshot in time in the course of the development of AD.

### Limitations of the study

The main limitation of the present study is that the data represent only a “snapshot” of AD prevalence and/or risk in a highly heterogenous group of former NFL players. As indicated in the Methods and Results sections, the subjects in this study played in the NFL at various time periods (1960–2010) and for different lengths of time (2–11 years) with a history of multiple concussions and various comorbidities. The imaging and neuropsychological data were collected at a single time point. Although none of the subjects in this study were diagnosed or treated for AD at the time of enrollment in the study, given that AD is the most common cause of dementia in patients presenting with dementia over the age of 65 years,^74^ brain scans of former NFL players were assessed only for AD pathology based on comprehensive volumetric analysis of brain regions that demonstrated bilaterally significant brain atrophy or *ex vacuo* dilatation in AD patients. However, the prevalence of frontotemporal lobar degeneration (FTLD) approximates that of AD in people under the age of 65.^75^ Therefore, it is possible that former NFL players in this study may develop other forms of dementia such as FTLD.

## Conclusion

The findings of our study lend credence to the reports that, next to aging, a history of TBI is the most important environmental risk factor for AD. Although there currently is no diagnostic test available for confirming which former NFL players will develop AD, the imaging markers highlighted in this study could potentially prove useful for predicting which contact athletes are at highest risk for developing AD.

## Abbreviations Used

3D: three-dimensional
AD: Alzheimer's disease
ADD: attention-deficit disorder
ADHD: attention-deficit hyperactivity disorder
ADNI: Alzheimer's Disease Neuroimaging Initiative
ALS: amyotrophic lateral sclerosis
ANAM4: Automated Neuropsychological Assessment Metric Version 4
CDS: code substitution-learning
CTE: chronic traumatic encephalopathy
fMRI: functional MRI
FTLD: frontotemporal lobar degeneration
HATA: hippocampal amygdala transition area
LH: left hemisphere
M2S: matching to sample
MPRAGE: magnetization-prepared rapid gradient echo
MRI: magnetic resonance imaging
mTBI: mild TBI
PD: Parkinson's disease
RH: right hemispphere
SD: standard deviation
ST6: Sternberg memory search
TBI: traumatic brain injury
TE: echo time
TR: repetition time
WRAT-IV: Wide Range Achievement Test-IV
